# Upgrading Care: Digital Cardiology Interventions to Reduce Risk∗

**DOI:** 10.1016/j.jacadv.2023.100590

**Published:** 2023-09-06

**Authors:** Neel P. Chokshi

**Affiliations:** a Department of Medicine, Perelman School of Medicine, University of Pennsylvania, Philadelphia, Pennsylvania, USA; b Penn Center for Digital Cardiology, University of Pennsylvania, Philadelphia, Pennsylvania, USA; c Penn Sports Cardiology & Fitness Program, University of Pennsylvania, Philadelphia, Pennsylvania, USA; d Leonard Davis Institute of Health Economics, University of Pennsylvania, Philadelphia, Pennsylvania, USA

**Keywords:** cardiac rehabilitation, coronary artery disease (CAD), digital health, remote patient monitoring

**Figure:**
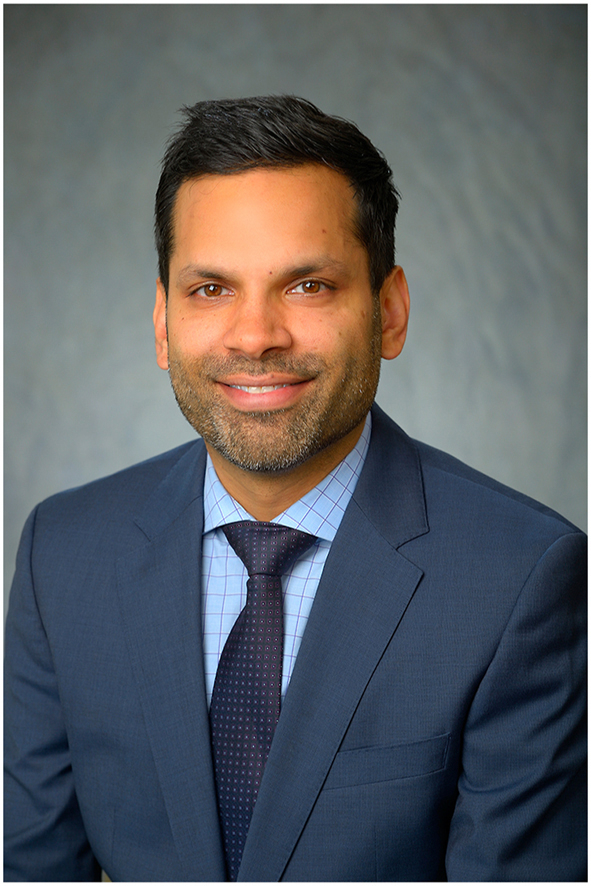

During the height of the COVID-19 pandemic, I met a new patient ‘in clinic’ via video consultation after an intensive care unit stay and discharge a few days earlier. He was a 62-year-old South Asian immigrant who presented with an acute myocardial infarction requiring percutaneous coronary intervention and hospital course complicated by coronavirus infection, pulmonary embolism, and cardiomyopathy. The patient was discharged home with remote monitoring using a tablet linked to a bluetooth-enabled blood pressure cuff, pulse oximeter, and scale. I assessed him weekly by telemedicine, conferencing both his adult children into video meetings from separate locations. Care coordination with his home nurses and therapists took place via the electronic health record. Eventually, we enrolled him in a virtual cardiac rehabilitation (CR) program to complete his recovery. There are numerous such anecdotes of tech-enabled care from the pandemic. Remote management of patients using digital health—telemedicine, mobile health, wearables, and information technology—has been exciting, informative, and at times trying. While these programs are gaining attention, there are limited data on the impact of these strategies on patient outcomes and costs. Further investigation is needed to guide meaningful design and large-scale implementation.

In this issue of *JACC: Advances*, Carrington et al^1^ review the evidence for digital health strategies to reduce cardiovascular risk. They performed a meta-analysis of digital health enabled disease management programs (DMPs) for patients after a coronary artery disease related hospitalization. The investigators pooled data from mostly randomized control trials comparing traditional postdischarge DMPs to care interventions utilizing elements of digital health technologies (or ‘mHealth’ as denoted by the authors). The primary outcomes were rates of hospital readmission and mortality. A patient DMP was defined as ‘a coordinated healthcare plan to help manage their disease better’. The majority of control interventions were traditional phase 2 CR programs including patient education and exercise guidance. Digital health interventions were broadly defined as DMPs implemented with ‘the use of wireless communication devices and/or software technology.’ These interventions were diverse including a combination of remote vitals and activity monitoring, texting interventions, and video consultations. The analysis included 18 trials and 3,818 patients spanning North America, Europe, and Australia from 2008 to 2021. Patient follow-up ranged from 1 to 24 months with most studies (n = 13) reporting outcomes for <6 months. The typical DMP intervention lasted 3 to 6 months.

Compared to traditional models of care, digital DMPs showed a 32% risk reduction in all-cause readmissions (RR: 0.68, 95% CI: 0.51-0.91, 96 fewer events), and a 45% risk reduction in cardiac readmissions (RR: 0.55, 95% CI: 0.44-0.68, 58 fewer events). The RR of emergency department visits was also reduced by 63% (RR: 0.37; 95% CI: 0.26-0.54, 42 fewer events). Digital DMPs did not differ with respect to patient mortality or major adverse cardiac events. The data here were constrained by inadequate evidence including small sample sizes and short follow-up duration.

There has been much consumer interest and business development around digital technologies to address cardiovascular health. Cardiology guidelines have also recently advocated for digital strategies to increase engagement and guide long-term behavioral change. While it is intuitive that technology provides unique opportunities to improve care delivery, large-scale and real-world evidence is limited. High startup costs and limited reimbursement for these services have been barriers to experimentation with digital DMPs. Importantly, this meta-analysis provides evidence that digital technologies significantly reduce resource utilization. The average 30-day readmission rate after a percutaneous coronary intervention in the United States is 12% with an average cost of approximately $13,000.^2^ In this cohort of approximately 3,800 patients, the reduction in 58 (2%) cardiac readmissions would roughly equate to $750,000 in hospitalization costs. Realization of a fraction of these savings would help to offset startup investments for payors and health systems. Additionally, operating at scale, well designed digital DMPs would create cost efficiencies (eg, lower real estate costs) compared to current models of care. In this work, all digital DMPs were remotely administered with some studies integrating elements of in-person care (9/18 studies, 50%) and some utilizing provider dashboards to monitor patient-generated data (10/18 studies, 56%). Such designs seem ideal for scalability, but research should explore their cost-effectiveness and the optimal balance of in-person and remote care.

Of course, future studies must also evaluate the impact on cardiac events and mortality. Even if digital DMPs are shown to be noninferior to traditional DMPs, this would demonstrate that they are a cost-saving alternative with additional potential advantages, such as patient satisfaction. Many digital health strategies, when designed with insights from behavioral science, have also shown promise for long-term behavior change such as increased physical activity.^3^ With reimbursement models transitioning towards value-based care, this is further incentive for health systems to invest in digital DMPs.

The study also highlights opportunities and risks related to health equity. Lower socioeconomic status is associated with reduced access to technology and the internet. During the pandemic, telemedicine utilization rates were lower among low-income, Black, and Latinx patients.^4^ In the current analysis, race and socioeconomic factors were not reported. Careful design and implementation are important to ensure that digital programs do not widen health disparities. The majority of digital DMPs in this study utilized texting (12/18 studies, 67%) for patient communication. In our experience, texting has broad reach and is easy to use, especially compared to portals.^5^ Additionally, mobile phones were the most common hardware component in studies (14/18 studies, 78%). Given the ubiquitous nature of mobile phones across socioeconomic groups, digital programs should focus on these devices. Additionally, cellular-enabled devices and internet hotspots should be incorporated as needed to ensure that all patients have access to digital programs.

Further, many of the interventions in both arms of the study consisted of traditional CR or overlapped with components of CR. CR is known to improve mortality and quality of life, but participation is limited. This is partly due to scarcity of centers, transportation, and patient costs, all of which are linked to socioeconomic status. The work here suggests that virtually implemented CR strategies may be beneficial in addressing these barriers. Future research should compare in-person and virtual CR to identify optimal strategies, including hybrid options, based on clinical scenarios.

Lastly, digital programs should leverage insights from behavioral economics to optimize patient engagement and long-term outcomes. ‘Goal setting’, ‘coaching’, and ‘self-management’ were all mentioned as components across both traditional and digital DMPs. Studies coupling digital health with strategies such as social incentives, gamification, and financial rewards have shown promise to reduce cardiovascular risk.^3^ Future work should ensure deliberate use of behavioral design.

In thinking back to that patient, I recall being a bit overwhelmed at the prospect of caring for such a sick patient entirely remotely. However, over time, I found many elements of his virtual care model to be more efficient than our traditional episodic, office-based care. When we finally did meet in person months later, I felt I had a more complete picture of his life, having virtually met his family and seen where he lived. Of course, meeting patients in person is still invaluable for the obvious reasons. Digital cardiology tools should not be a replacement for patient visits but rather serve to augment the patient-clinician relationship. Hopefully, in time, we will generate data to confirm that these tools translate into improved outcomes.

Understanding the right patient, the appropriate scenario, and the relevant technology will be critical and should be the focus of research moving forward. We will also need to examine the impact on the patient experience and clinician workloads. To ensure meaningful improvement in care, digital cardiology approaches should be rooted in thoughtful design and integration of evidence where feasible (Figure 1). Implementation will require significant team-based efforts and financial investment, but there is considerable potential to improve patient care on a large scale.

**Figure 1:**
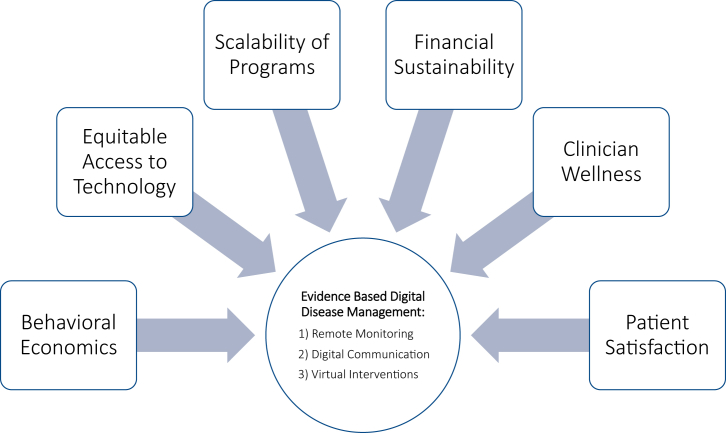
**Guiding Principles for Future Design of Digital CardiologyPrograms**

## Funding support and author disclosures

The author has reported that he has no relationships relevant to the contents of this paper to disclose.
